# Axillary Lymph Node Dissection Rates and Prognosis From Phase III Neoadjuvant Systemic Trial Comparing Neoadjuvant Chemotherapy With Neoadjuvant Endocrine Therapy in Pre-Menopausal Patients With Estrogen Receptor-Positive and HER2-Negative, Lymph Node-Positive Breast Cancer

**DOI:** 10.3389/fonc.2021.741120

**Published:** 2021-09-30

**Authors:** Sungchan Gwark, Woo Chul Noh, Sei Hyun Ahn, Eun Sook Lee, Yongsik Jung, Lee Su Kim, Wonshik Han, Seok Jin Nam, Gyungyub Gong, Seon-Ok Kim, Hee Jeong Kim

**Affiliations:** ^1^ Department of Surgery, College of Medicine, Asan Medical Center, University of Ulsan, Seoul, South Korea; ^2^ Department of Surgery, Korea Cancer Center Hospital, Korea Institute of Radiological and Medical Sciences, Seoul, South Korea; ^3^ Department of Surgery, Center for Breast Cancer, Research and Institute and Hospital, National Cancer Center, Goyang, South Korea; ^4^ Department of Surgery, School of Medicine, Ajou University, Suwon, South Korea; ^5^ Division of Breast and Endocrine Surgery, College of Medicine, Hallym Sacred Heart Hospital, Hallym University, Anyang, South Korea; ^6^ Department of Surgery and Cancer Research Institute, College of Medicine, Seoul National University, Seoul, South Korea; ^7^ Department of Surgery, Samsung Medical Center, School of Medicine, Sungkyunkwan University, Seoul, South Korea; ^8^ Department of Pathology, College of Medicine, Asan Medical Center, University of Ulsan, Seoul, South Korea; ^9^ Department of Clinical Epidemiology and Biostatistics, Asan Medical Center, Seoul, South Korea

**Keywords:** axillary lymph node dissection, survival, prognosis, neoadjuvant chemotherapy, neoadjuvant endocrine therapy, neoadjuvant study of chemotherapy *versus* Endocrine therapy in premenopausal patient with hormone responsive, HER2-negative, lymph node-positive breaST (NEST)

## Abstract

In this study, we aimed to evaluate axillary lymph node dissection (ALND) rates and prognosis in neoadjuvant chemotherapy (NCT) compare with neoadjuvant endocrine therapy (NET) in estrogen receptor-positive (ER+)/human epidermal growth factor receptor 2-negative (HER2-), lymph node (LN)-positive, premenopausal breast cancer patients (NCT01622361). The multicenter, phase 3, randomized clinical trial enrolled 187 women from July 5, 2012, to May 30, 2017. The patients were randomly assigned (1:1) to either 24 weeks of NCT including adriamycin plus cyclophosphamide followed by intravenous docetaxel, or NET involving goserelin acetate and daily tamoxifen. ALND was performed based on the surgeon’s decision. The primary endpoint was ALND rate and surgical outcome after preoperative treatment. The secondary endpoint was long-term survival. Among the 187 randomized patients, pre- and post- neoadjuvant systemic therapy (NST) assessments were available for 170 patients. After NST, 49.4% of NCT patients and 55.4% of NET patients underwent mastectomy after treatment completion. The rate of ALND was significantly lower in the NCT group than in the NET group (55.2% *vs.* 69.9%, P=.046). Following surgery, the NET group showed a significantly higher mean number of removed LNs (14.96 *vs.* 11.74, P=.003) and positive LNs (4.84 *vs.* 2.92, P=.000) than the NCT group. The axillary pathologic complete response (pCR) rate was significantly higher in the NCT group (13.8% *vs.* 4.8%, P=.045) than in the NET group. During a median follow-up of 67.3 months, 19 patients in the NCT group and 12 patients in the NET group reported recurrence. The 5-year ARFS (97.5%*vs.* 100%, P=.077), DFS (77.2% *vs.* 84.8%, P=.166), and OS (97.5% *vs.* 94.7%, P=.304) rates did not differ significantly between the groups. In conclusion, although survival did not differ significantly, more NCT patients might able to avoid ALND, with fewer LNs removed with lower LN positivity.

**Clinical Trial Registration:**

https://clinicaltrials.gov/ct2/show/NCT01622361, identifier NCT01622361.

## Introduction

In the post-surgery management of patients with breast cancer, lymphedema is the surgical morbidity surgeons are most likely to encounter and prefer to avoid. Decision-making on axillary treatment among patients undergoing neoadjuvant systemic therapy (NST) has become increasingly complex. For patients with clinically node-negative breast cancer, the application of the American College of Surgeons Oncology Group (ACOSOG) Z0011 trial criteria (1, 2) in women undergoing upfront breast-conserving surgery (BCS) and the use of NST to downstage microscopic axillary disease are among the viable options to reduce the necessity for axillary lymph node dissection (ALND) (3–5).

Although NST is associated with the potential for axillary nodal downstaging, the rates of nodal pathologic complete response (pCR) differ substantially by tumor subtype, the rate being higher in human epidermal growth factor receptor 2 (HER2)-positive and triple-negative breast cancers (6–9). However, in patients with the estrogen receptor-positive (ER+)/HER2- subtype, the rate is relatively low (10–13). Considering these features, the American Society of Clinical Oncology (ASCO) guideline suggests that neoadjuvant chemotherapy (NCT) may be administered to ER+/HER2- patients if the tumor stage is such that chemotherapy will be administered regardless of surgical timing. In this case, the same regimen should be followed as would be considered after surgery (14, 15). Pilewskie et al. suggested different strategies to minimize the ALND rates in patients with node-negative, early-stage breast cancer with differing tumor biology (16). However, the use of such strategies for ER+/HER2-, especially for lymph node-positive (LN+) breast cancer, has not been adequately investigated, and it remains difficult to ascertain the appropriate strategies.

We conducted a clinical trial of neoadjuvant chemotherapy (NCT) versus neoadjuvant endocrine therapy (NET) in premenopausal patients with hormone-responsive, HER2-, LN+ breast cancer. As we previously reported, in a phase III trial (NEST; NCT01622361), conventional NCT yielded a significantly better response than NET in premenopausal patients with ER+/HER2-, LN+ breast cancer, supporting the ASCO and St. Gallen international consensus guidelines (15, 17, 18). The present study aimed to evaluate the surgical impact of neoadjuvant treatment and compare the ALND rates and prognosis in terms of axillary recurrence-free survival (ARFS), disease-free survival (DFS), and overall survival (OS) between patients treated with NET and NCT in the NEST trial.

## Methods

### Study Design and Participants

NEST was a prospective, multicenter, randomized, parallel group, comparative phase III clinical trial. Seven centers in the Korean Breast Cancer Society Group participated (KBCSG-012). The study was approved by the Korea Food and Drug Administration (KFDA). Approval was granted by the institutional review board at each trial center. The trial protocol is summarized in **Supplement 1**. This study followed the Consolidated Standards of Reporting Trials reporting guidelines. The detailed study protocol was published in 2020 (17).

Premenopausal women with histologically confirmed ER+/HER2-, LN+ primary breast cancer were eligible for the study. Histologically proven LN positivity was necessary before initiating treatment with core needle biopsy or fine needle aspiration. The study participants were 20–50 years in age. Premenopausal status was defined based on the following criteria: last menses occurring within 6 months prior to randomization and previous hysterectomy, estradiol levels ≥20 pg/ml, and follicle-stimulating hormone level <30 mIU/ml within 4 weeks prior to randomization.

Pathological specimens were assessed in each institutional laboratory. ER positivity was defined as an Allred score ≥3 or modified Allred score ≥4. The HER2 status was confirmed as negative if the immunohistochemistry score was 1+, or if the score was 2+ and the result of fluorescence or silver *in situ* hybridization for HER2 amplification was negative (19). Patients with inflammatory breast cancer, bilateral breast cancer, evidence of distant metastasis, or other malignancies were excluded. Written informed consent was obtained from all participants.

### Procedures

**Figure 1:**
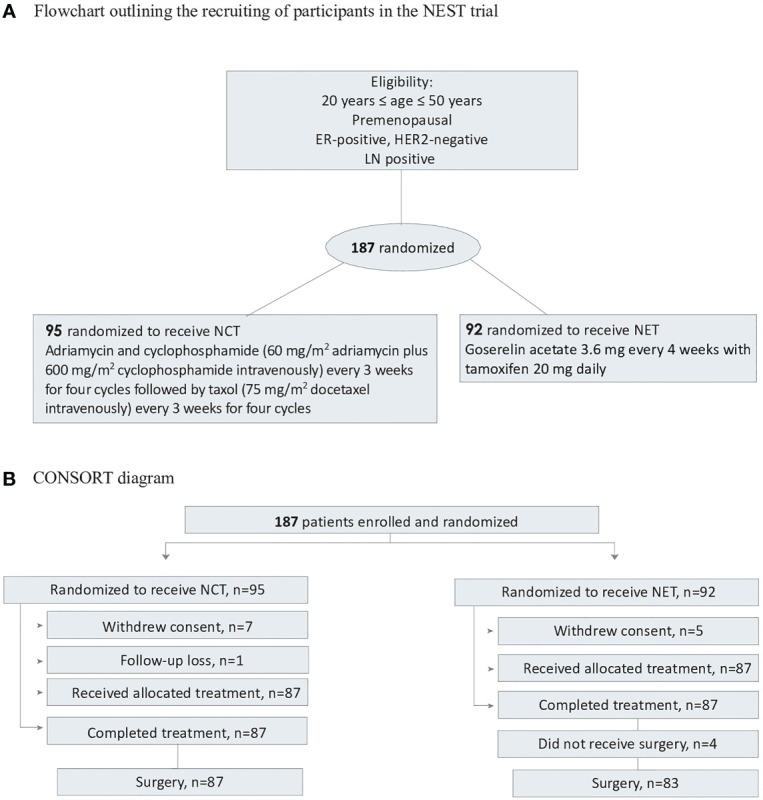
Flowchart and CONSORT diagram. **(A)** Flowchart outlining the recrutinng of participants in the NEST trial. **(B)** ConSort diagram. ER, estrogen; HER, human epidermal grtoeth factor receptor 2; NCT, neoadjuvant chemotherapy; NET, neoadjuvant endocrine therapy.

Patients were randomly assigned (1:1) to receive either NCT or endocrine therapy for 24 weeks prior to surgery. The patients were stratified by the treatment center and clinical stage (stages II and III). Results of the treatment arm, which have been previously published, are shown in **Supplement 1**. Patients were randomly assigned to either receive 60 mg/m^2^ of adriamycin plus 600 mg/m^2^ of cyclophosphamide intravenously every 3 weeks for four cycles followed by 75 mg/m^2^ of docetaxel intravenously every 3 weeks for four cycles, or to receive goserelin acetate 3.6 mg every 4 weeks with tamoxifen 20 mg daily. Treatment continued for 24 weeks before surgery (**Figure 1**).

All patients underwent breast magnetic resonance imaging (MRI) before the start of treatment and after the completion of treatment, prior to surgery.

Surgery was performed between weeks 24 and 26. Sentinel node biopsy (SNB) procedure was performed for all patients. For identification of the sentinel lymph node, all participating centers used radioisotope (Tc99m), blue dye, or a combination of these methods. Axillary surgery was performed as considered appropriate by each surgeon. Patients with residual positive lymph node in sentinel lymph node biopsy received either conventional ALND or axillary sampling at the discretion of the surgeon. The axillary sampling was defined as the removal of several axillary lymph nodes located near the sentinel lymph nodes without full exposure of the surrounding structures such as axillary vein, long thoracic nerve, and thoracodorsal nerve. To minimize the risk of erroneous classification of the axilla, ALND was defined according to previous studies as anatomic level I and II dissection including at least 10 lymph nodes (20–24).

### Outcomes

The primary outcome measure for present surgical study was ALND rate, defined as the rate of removal of >10 axillary LNs in levels 1 and 2 after the completion of NST. Secondary outcome was survival analysis, which included ARFS, DFS, and OS.

### Statistical analyses

The sample size was calculated based on the clinical response rate measured by MRI in the NCT and NET groups under the assumption that the effect of NET would be non-inferior to that of NCT. Detailed description for sample size calculation in the original study has been published previously (17). Data were analyzed from May 1 to October 31, 2020. Data were summarized based on frequency and percentage for categorical variables and mean and standard deviation for continuous variables. Differences between the NET and NCT groups were evaluated by the Student’s *t*-test or Mann-Whitney test for continuous variables and the chi-square or Fisher’s exact tests for categorical variables. The ALND rate between the NET and NCT groups was compared using the chi-square test. Survival rates were estimated using the Kaplan–Meier method and compared using the log-rank test. Statistical analyses were conducted using SPSS Statistics version 26.0 for Windows (IBM Corp., Armonk, NY, USA). P-value <0.05 was considered significant.

## Results

A total of 194 patients from seven participating centers were enrolled between July 5, 2012 and September 24, 2014; 7 patients discontinued and 187 were randomized. Seven patients in the NCT group and five patients in the NET group withdrew their consent. One patient in the NCT group was randomized but did not receive treatment. The remaining 174 patients were randomly assigned to receive chemotherapy (n=87) or endocrine therapy (n=87). All patients completed the 24-week neoadjuvant treatment course. After completion of the randomly assigned preoperative treatment, four patients in the NET group refused to undergo surgery (3 patients showed partial response and 1 patient showed stable disease). Eventually, 170 patients were studied. Adjuvant radiotherapy was homogeneously administered in the two groups which was indicated in all BCS patients and in mastectomy patients with large tumors (5cm or larger), four or more positive lymph nodes, or positive margins. Patient and tumor characteristics are outlined in **Table 1**.

**Table 1:** Clinicopathological characteristics of the treatment groups^a,b^.

Variable	NCT (n = 87)	NET (n = 83)	*P*
**Age at diagnosis, years**			
Mean (SD)	42.5 ± 5.6	41.7 ± 5.7	.366
20–29	2 (2.3)	1 (1.2)	
30–39	20 (23.0)	29 (34.9)	
40–49	60 (69.0)	51 (61.4)	
50–55	5 (5.7)	2 (2.5)	
**Histology**			.590
Ductal	79 (90.8)	76 (91.6)	
Lobular	5 (5.7)	6 (7.2)	
Other^c^	3 (3.5)	1 (1.2)	
**Clinical T stage**			.906
cT1	18 (20.7)	14 (16.9)	
cT2	54 (62.1)	54 (65.1)	
cT3	14 (16.1)	14 (16.9)	
cT4	1 (1.1)	1 (1.1)	
**Grade**			.961
G1/2	65 (75.6)	62 (75.6)	
G3	5 (5.8)	4 (4.9)	
N/A	16 (18.6)	17 (20.5)	
**PR status** ^d^			.911
Positive	77 (88.5)	73 (88.0)	
Negative	10 (11.5)	10 (12.0)	
**Ki67 expression**			.874
<20	32 (37.6)	30 (36.1)	
≥20%	53 (62.4)	53 (63.9)	
**Breast surgery**			.447
BCS	44 (50.6)	37 (44.6)	
Mastectomy	43 (49.4)	46 (55.4)	

a Unless otherwise indicated, data are expressed as number (percentage) of patients.

b Clinical N stage was excluded due to the heterogeneous assessment of patients during physical examination.

c Other histologies include invasive micropapillary (n = 4), mucinous (n = 10), and invasive tubular (n = 1) carcinomas.

d All patients were ER-positive.

BCS, breast-conserving surgery; cT1, clinical T1; cT2, clinical T2; cT3, clinical T3; NCT, neoadjuvant chemotherapy; NET, neoadjuvant endocrine therapy; PR, progesterone receptor.

The median age was 42 years (range, 27–54 years). All patients were premenopausal. Sixty-five percent of patients had clinical T2 breast cancer. Ninety-four percent of patients had G1/2 breast cancer. Few patients (<5%) had poorly differentiated (G3) tumor. The mean Ki-67 expression did not differ between the two groups (26.3 for NCT *vs.* 26.7 for NET, *P*=.874). As shown in **Table 1**, 49.4% of NCT patients and 55.4% of NET patients underwent mastectomy after treatment completion and the difference was not statistically significant (*p* = 0.447). Seven patients (8.0%) in the NCT group and one patient (1.2%) in the NET group achieved pCR. Nine patients (10.3%) in the NCT group and one patient (1.2%) in the NET group achieved pCR in the breast. The axillary pCR rate was significantly higher in the NCT group (13.8% *vs.* 4.8%, *P*=.045). The NCT group showed a significantly lower ALND rate than the NET group (56.3% *vs.* 71.1%, *P*=.046) after neoadjuvant therapy. Furthermore, the NCT group showed fewer LNs removed (mean, 11.74 *vs.* 14.96, *P*=.003), with lower LN positivity (mean, 2.92 *vs.* 4.84, *P*=.000) compared to the NET group (**Table 2**). When grouped by type of surgical management, the mastectomy group demonstrated a significantly higher rate of ALND and higher mean number of removed axillary lymph nodes compared to BCS group (**Supplement 2**).

**Table 2:** Comparison of pathological response and axillary lymph node results by treatment group^a^.

	NCT (%)	NET (%)	*P*
**Complete pCR**	7 (8.0)	1 (1.2)	.064
**Breast pCR**	9 (10.3)	1 (1.2)	.018
**Axillary pCR**	12 (13.8)	4 (4.8%)	.045
**Number of removed axillary LNs**			
<10 (SNB only or AS)	38 (43.7)	24 (28.9)	.046
≥10 (ALND^b^)	49 (56.3)	59 (71.1)	
**Mean number of removed axillary LNs (SD)**	11.74 ± 6.6	14.96 ± 7.2	.003
**Mean number of positive axillary LNs (SD)**	2.92 ± 3.9	4.84 ± 4.7	.000

a Unless otherwise indicated, data are expressed as number (percentage or standard deviation, SD) of patients.

b Axillary lymph node dissection: Number of removed axillary lymph nodes ≥10 in levels 1 and 2.

AS, axillary sampling; NCT, neoadjuvant chemotherapy; NET, neoadjuvant endocrine therapy; LNs, lymph nodes; pCR, pathologic complete response; SNB, sentinel lymph node biopsy.

**Figure 2:**
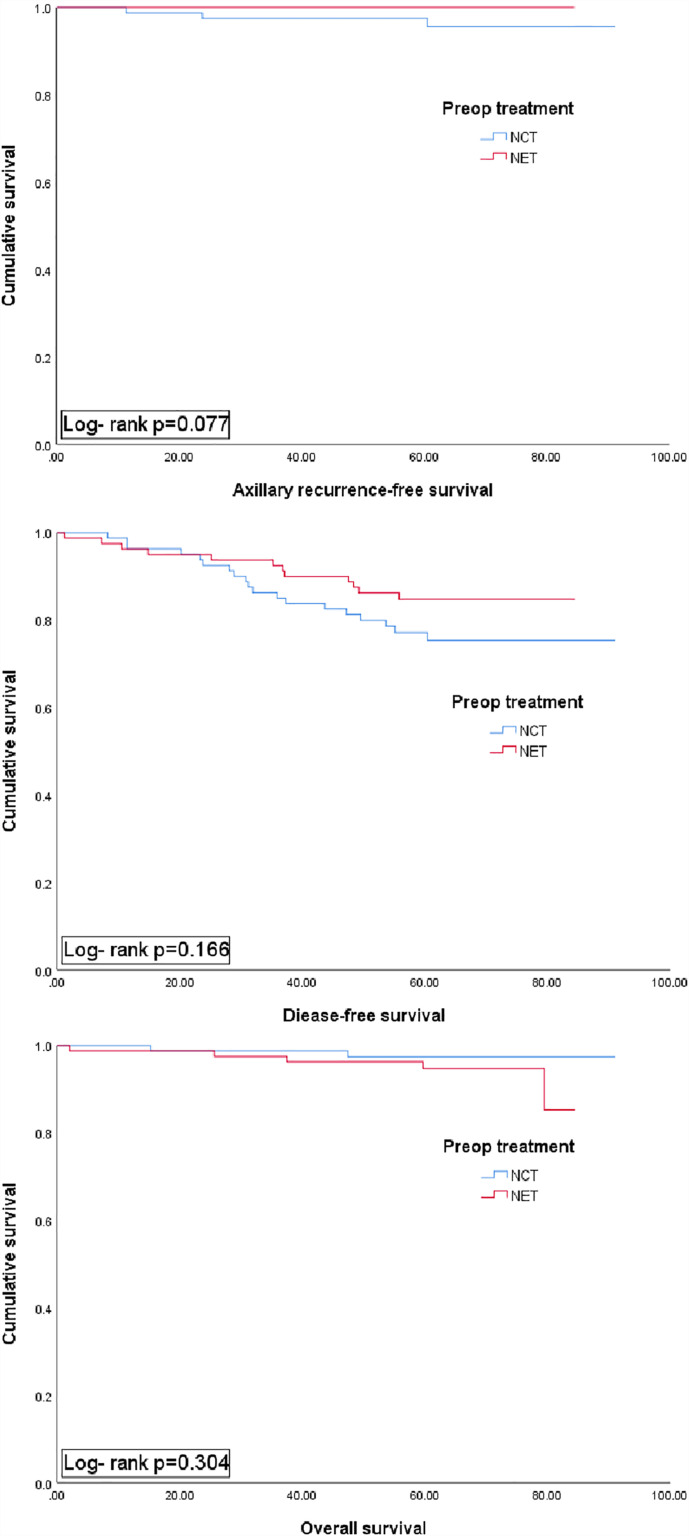
Kaplan-Meier plots for axillary recur-free survival, disease free surviaval and overall survival accordibg to preoperative (preop) treatment group (NCT *vs.* NET). NCT, neoadjuvant chemotherapy; NET, neoadjuvant endocrine therapy.

During a median follow-up of 67.3 months, recurrence occurred in 19 patients in the NCT group (local, n=3; axillary, n=3; regional; internal mammary LN recurrence, n=1; distant metastasis, n=12) (**Supplement 2**) and 12 patients in the NET group (all distant metastasis, n=12). A Kaplan–Meier survival analysis revealed no statistically significant differences in 5-year ARFS (97.5% *vs.* 100%, *P*=.077), DFS (77.2% *vs.* 84.8%, *P=*.166) and 5-year OS (97.5% *vs.* 94.7%, *P=*.304) between the NCT and NET groups (**Figure 2**).

## Discussion

In the NEST trial, ALND was avoidable in a greater proportion of patients who received NCT than in those who received NET. This suggests that one of the primary purposes of NST in HR+/HER2- breast cancer, in which response to NCT is unrelated to survival, might be de-escalation of axillary surgery. To the best of our knowledge, this is the first study to compare the surgical impact of NCT and NET in premenopausal women with breast cancer. Additionally, this study is unique because all patients had ER+/HER2- tumors and pathologically proven LN+ disease.

Unlike in patients with the triple-negative or HER2-positive subtype, clinical response and pCR after NCT are not reported as surrogate end points for long-term outcomes in patients with ER+/HER2- tumors, even in those with node-positive disease (25). Breast cancer survival is relatively higher in this subtype of patients, regardless of pCR achievement. The rate of pCR was shown to be lowest in patients with ER+/HER2-tumors, and achievement of pCR may not be a prognostic factor for survival for this subtype (25–27). These findings are consistent with those from our study, in which the NCT group achieved significantly higher axillary pCR, with no difference observed in ARFS.

Despite the limitations of NCT in ER+/HER2- tumors, this treatment could reduce the need for ALND in patients with negative conversion of initial metastatic LNs. Avoidance of ALND could improve the post-surgical quality of life for patients, because surgical morbidity is substantially less after sentinel node biopsy (SNB) alone than after ALND, with significantly lower rates of lymphedema, sensory changes, wound infection, and arm dysfunction reported (28–30). The technical feasibility of SNB alone after NST for LN+ breast cancer patients was established in three multicenter trials (31–33) that evaluated the identification and false-negative rates of SNB after NCT among clinically node-positive patients. The trials reported acceptable (<10%) when three or more sentinel nodes were retrieved. The ACOSOG Z1071 (Alliance) trial included 649 patients (cT0-4 cN1-2 M0) (32) and the SN FNAC study included 153 patients (cT0-3 cN1-2) (33). In both protocols, SNB and ALND were performed for all patients after NCT. The SENTINA trial was a multi-arm study including 592 patients (cN1-2), which included patients who converted to clinical N0 (determined by physical examination) after NCT and underwent SNB and ALND (31). The detection rate (identification of at least one sentinel lymph node (SLN) in these studies ranged from 80% to 93%. The rates for identification of three or more SLNs were variable, ranging from only 34% in the SENTINA trial to 57% in the Alliance trial (32), and as high as 86% in a recent cohort of 128 patients (cT0-3 cN1) from the Memorial Sloan Kettering Cancer Center (MSKCC) who converted to clinical N0 after NCT (13). Kang et al. showed that in patients with breast cancer with axillary LN conversion from clinically positive to negative status following NCT, the SNB-guided axillary operation and ALND without SNB led to similar rates of axillary and distant recurrence (34).

Although SNB has been adopted to allow the de-escalation of local therapy following NST, long-term prognostic analyses for those achieving an axillary pCR by SNB only are limited. Currently available evidence includes that from a single institution retrospective series reported by Galimberti et al., where a subgroup of 70 clinically node-positive patients treated with NCT, who converted to clinically node-negative status following treatment and underwent negative SNB, demonstrated no axillary recurrences at a median follow-up of 61 months (35). The authors noted that chest-wall and/or regional nodal radiotherapy might be particularly important in clinically node-positive patients before neoadjuvant therapy, which is currently being evaluated in two large randomized trials. The NSABP B-51/RTOG 1304 trial will confirm the oncologic safety of SNB alone in women with clinically node-positive disease, who have negative axillary staging and are randomized to regional nodal irradiation versus no further axillary therapy (36). The Alliance A11202 trial will study a population of women with positive sentinel nodal disease, evaluating whether ALND can be avoided in favor of regional nodal irradiation in this population. Both trials will help address important issues related to tailoring local treatment based on the extent of nodal disease in women undergoing neoadjuvant treatment.

Our study showed that among 170 patients with biopsy-proven nodal metastases, 37.6% (n=64) became eligible for avoiding ALND following neoadjuvant therapy, similar to the observation in the MSKCC cohort (13). The rate was higher in the NCT group (38/87, 43.7%) than the NET group (24/83, 28.9%), and axillary pCR was also significantly higher in the NCT group than the NET group (13.8% *vs.* 4.8%, *P*=.045). Therefore, NCT could be a better treatment strategy than NET to avoid LN dissection. Although, BCS rate and locoregional recur was higher in NCT group, compared to those who receive NET, this difference was not statistically significant (p = 0.447, **Table 1**) and neither surgical treatment nor radiotherapy was related to local recurrence (**Supplement 3**). In our previous report, the NCT group showed better response to neoadjuvant treatment (17), however, we presently observed no significant differences in the 5-year ARFS, DFS, and OS between the NCT and NET groups. The current findings support those of previous studies stating that better response to NST does not guarantee better prognosis, especially in the ER+/HER- tumor subtype (25–27).

Currently, limited data are available on NET in premenopausal women, because most NET studies in breast cancer have focused on postmenopausal women (37–40). Some studies have shown that NET could be effective in a cohort of well-selected premenopausal patients (41, 42). The Grupo Español de Investigación en Cáncer de Mama (GEICAM) reported the randomized phase II results of chemotherapy versus exemestane in pre- and postmenopausal women (43). Although the sample size was small, the response rate was higher for chemotherapy than for endocrine therapy in premenopausal patients, which is consistent with the findings reported in our study. In our study, the patients received 6 months of NET; however, the optimal duration of NET was not defined appropriately. Most NET studies performed previously involved 3 to 6 months of therapy. In a study by Llombart-Cussac et al. (40), 37% of patients achieved the maximal response beyond 6 months. Carpenter et al. showed that the median time to achieve BCS (in those who responded) was 7.5 months (44). Notably, 62% of panelists at St Gallen 2013 were in favor of continuing NET until a maximal response was achieved (45). If treatment was continued until maximal response was achieved, the response in our study may have been better. In this study, axillary pCR rate after NET was relatively lower than that after NCT. For those who carry residual nodal burden after NET, whether ALND should be performed is a challenging issue. Kantor et al. (46) demonstrated no differences in 5-year OS between patients with axillary pCR and those with any residual nodal disease category after NET. The results suggest that unlike NCT patients, the outcomes of NET patients mirror those of upfront surgery patients. This presents an opportunity to consider de-escalation of axillary management strategies in NET patients. Additionally, the lack of survival difference in upfront surgery trials of alternative axillary management strategies, including the Z0011 (2, 47) and AMAROS (48) trials, suggests an opportunity for the de-escalation of axillary surgery in patients treated with NET. Furthermore, unlike NCT, patients who fail to achieve axillary pCR after NET are eligible to receive adjuvant chemotherapy, which could control loco-regional and systemic recurrence (49). Thus, while further studies are needed, the adoption of axillary management strategies utilized in upfront surgery patients, rather than in NCT patients, may be more appropriate in patients receiving NET.

Our study has several limitations. First, the sample size was small and did not satisfy the predefined number. Second, although the main study is a phase 3 clinical trial, the field of investigation in this paper has a retrospective nature, as we have classified the axillary procedure according to post-op pathologic results of the patients. Third, we did not include an aromatase inhibitor as an NET treatment arm. In a study that compared the effects of neoadjuvant gonadotropin releasing hormone (GnRH) analog plus tamoxifen and GnRH analog plus anastrozole in premenopausal women, the clinical response was better in the anastrozole group (42). As we did not consider an aromatase inhibitor, the response comparison between chemotherapy versus ovarian function suppression using aromatase inhibitor in premenopausal women remains incomplete. Fourth, patients receiving NET received only a small portion of their overall course of endocrine therapy. Finally, ALND was performed based on the decision of each surgeon, since there are no unified guidelines available currently. This was highlighted in the study performed by Morrow et al. (50), in which data regarding the attitude, decision-making, or acceptance of limited axillary surgery among surgeons were reported, which were shown to vary widely. Additionally, the relatively short follow-up period for the assessment of prognosis in ER+/HER2- patients limited the statistical power of the prognostic results.

In conclusion, a greater proportion of patients who receive NCT might be able to avoid ALND, which could result in the removal of fewer LNs with lower LN positivity as well as a higher pCR achievement rate, compared to those who receive NET, especially among young women with ER-positive/HER2-negative and LN+ breast cancer. However, while further studies are needed, for patients treated with NET, especially those with residual nodal burden, the adoption of axillary management strategies utilized in upfront surgery patients rather than in NCT patients, may be more appropriate which could lead to the de-escalation of axillary surgery. Although no significant differences were observed between the two groups in terms of ARFS, DFS, and OS, further analyses with longer follow-up data are warranted to re-assess long-term survival in these patients.

## Data Availability Statement

The raw data supporting the conclusions of this article will be made available by the authors, without undue reservation.

## Ethics Statement

The studies involving human participants were reviewed and approved by Asan Medical Center Institutional Review Board. The patients/participants provided their written informed consent to participate in this study.

## Author Contributions

SA had full access to all of the data in the study and HK takes responsibility for the integrity of the data and the accuracy of the data analysis. HK designed the study. SG and HK drafted the manuscript. HK wrote the original protocol for the study. All authors participated in the design of the study. HK filed for ethical approval from KFDA and registered the trial on Clinicaltrials.gov. GG was responsible for the pathology reports. S-oK performed the statistical analyses. SA conceived the study and participated in its design. SA, WN, EL, YJ, LK, WH, and SN were involved in the study design and inclusion of patients in the trial. All authors contributed to the article and approved the submitted version.

## Funding

This study was sponsored by AstraZeneca Korea Ltd. The funder was not involved in the study design, collection, analysis, interpretation of data, the writing of this article or the decision to submit it for publication.

## Conflict of Interest

The authors declare that the research was conducted in the absence of any commercial or financial relationships that could be construed as a potential conflict of interest.

## Publisher’s Note

All claims expressed in this article are solely those of the authors and do not necessarily represent those of their affiliated organizations, or those of the publisher, the editors and the reviewers. Any product that may be evaluated in this article, or claim that may be made by its manufacturer, is not guaranteed or endorsed by the publisher.
