# Supplementary material for: Axillary Lymph Node Dissection Rates and Prognosis From Phase III Neoadjuvant Systemic Trial Comparing Neoadjuvant Chemotherapy With Neoadjuvant Endocrine Therapy in Pre-Menopausal Patients With Estrogen Receptor-Positive and HER2-Negative, Lymph Node-Positive Breast Cancer
Source: Front Oncol. 2021 Sep 30;11:741120. doi: 10.3389/fonc.2021.741120 (PMC8515848; doi:10.3389/fonc.2021.741120)
Supplement: Supplementary file 1 [file DataSheet_1.docx]

**Supplement Figure 1.** Kaplan–Meier plots for axillary recur-free survival according to surgical management and adjuvant radiotherapy in NCT group.


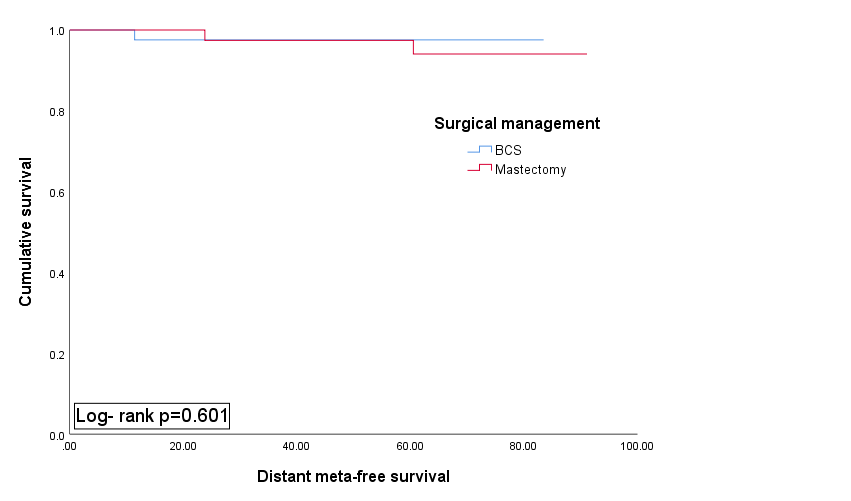


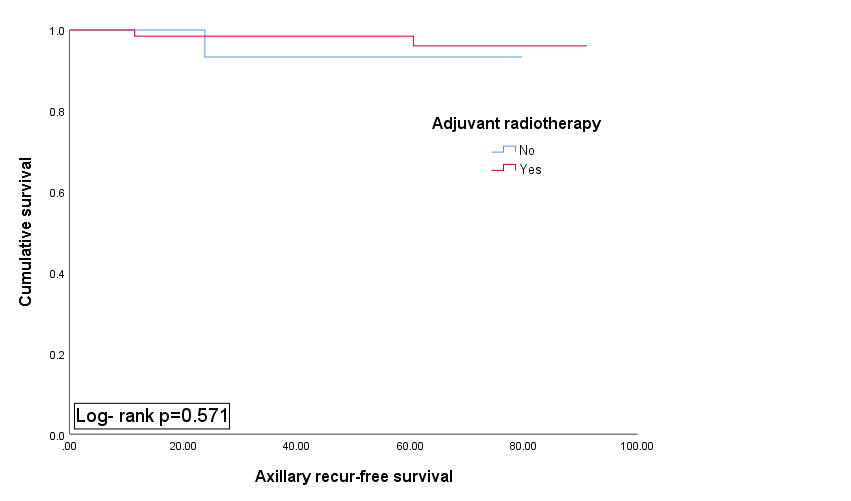


BCS, breast conserving surgery; NCT, neoadjuvant chemotherapy
