# Supplementary material for: Axillary Lymph Node Dissection Rates and Prognosis From Phase III Neoadjuvant Systemic Trial Comparing Neoadjuvant Chemotherapy With Neoadjuvant Endocrine Therapy in Pre-Menopausal Patients With Estrogen Receptor-Positive and HER2-Negative, Lymph Node-Positive Breast Cancer
Source: Front Oncol. 2021 Sep 30;11:741120. doi: 10.3389/fonc.2021.741120 (PMC8515848; doi:10.3389/fonc.2021.741120)
Supplement: Supplementary file 2 [file Table_1.docx]

**Supplement Table 1.** Comparison of axillary surgery by type of surgical management^a^

|  | BCS (%) | Mastectomy (%) | *P* |
| --- | --- | --- | --- |
| **Number of removed axillary LNs (range)**  <10 (SNB only or AS) | (3-26) | (1-31) |  |
|  | 36 (44.4) | 26 (36.5) | .039 |
| ≥10 (ALND^b^) | 45 (55.6) | 63 (63.5) |  |
| **Mean number of removed axillary LNs (SD)** | 11.74±6.5 | 14.52±7.5 | .011 |
| **Mean number of positive axillary LNs (SD)** | 3.01±3.4 | 4.63±5.1 | .015 |

^a^Unless otherwise indicated, data are expressed as number (percentage or standard deviation, SD) of patients.

^b^Axillary lymph node dissection: Number of removed axillary lymph nodes ≥10 in levels 1 and 2

AS, axillary sampling; NCT, neoadjuvant chemotherapy; NET, neoadjuvant endocrine therapy; LNs, lymph nodes; pCR, pathologic complete response; SNB, sentinel lymph node biopsy
