# Supplementary material for: Axillary Lymph Node Dissection Rates and Prognosis From Phase III Neoadjuvant Systemic Trial Comparing Neoadjuvant Chemotherapy With Neoadjuvant Endocrine Therapy in Pre-Menopausal Patients With Estrogen Receptor-Positive and HER2-Negative, Lymph Node-Positive Breast Cancer
Source: Front Oncol. 2021 Sep 30;11:741120. doi: 10.3389/fonc.2021.741120 (PMC8515848; doi:10.3389/fonc.2021.741120)
Supplement: Supplementary file 3 [file Table_2.docx]

**Supplement Table 2.** Locoregional recurrences of NCT group

| Age | Histology | clinical stage | PR | Surgical management | Pathologic stage | Radiation therapy | Location  of recurrence | Status at last follw-up |
| --- | --- | --- | --- | --- | --- | --- | --- | --- |
| 44 | IDC | cT2N1 | + | BCS+ALND | ypT2N1 | yes | Local | alive with disease |
| 49 | IDC | cT3N2 | - | Mastectomy+SNB | ypT1N1 | yes | Local | alive with disease |
| 50 | IDC | cT2N1 | + | BCS+ALND | ypT1N1 | yes | Local | alive with disease |
| 29 | IDC | cT2N2 | + | BCS+ALND | ypT2N1 | yes | ALN | dead of disease |
| 42 | Mucinous carcinoma | cT2N1 | + | Mastectomy+SNB | ypT2N1 | no | IMLN | alive with disease |
| 44 | IDC | cT2N1 | + | Mastectomy+AS | ypT2N1 | no | ALN | alive with disease |
| 45 | IDC | cT3N1 | + | Mastectomy+SNB | ypT2N1 | yes | ALN | alive with disease |

ALN, axillary lymph node; AS, axillary sampling; IDC, invasive ductal carcinoma; IMLN, internal mammary lymph node; NCT, neoadjuvant chemotherapy;
